# Statin Withdrawal Beyond Acute Phase Affected Outcome of Thrombolytic Stroke Patients: An Observational Retrospective Study

**DOI:** 10.1097/MD.0000000000000779

**Published:** 2015-05-01

**Authors:** Lu-sha Tong, Hai-tao Hu, Sheng Zhang, Shen-qiang Yan, Min Lou

**Affiliations:** From the Department of Neurology, The 2nd Affiliated Hospital of Zhejiang University, School of Medicine, Hangzhou, China.

## Abstract

Statin withdrawal is associated with deleterious outcome on stroke patients. Whether risk changes over time, depends on concomitant treatment of intravenous thrombolysis, or both remains to be clarified. We assessed the influence of statin withdrawal within 3 weeks while initiated in acute phase (72 hours) among patients receiving intravenous thrombolysis.

This was a monocentered retrospective observational study enrolling intravenous thrombolytic stroke patients from June 2009 to May 2014. Consecutive patients were distinguished into 3 groups according to the initiation and withdrawal of statin: the reference group (not received statin in 72 hours after stroke onset); the continued group (initiated statin therapy in 72 hours and continued for at least 3 weeks); the withdrawal group (initiated statin in 72 hours and discontinued within 3 weeks). All reasons for cessation were recorded. The effects of statin withdrawal on short-, mid-, and long-term outcomes were evaluated as neurologic improvement (NIH Stroke Scale [NIHSS] score improvement ≥4 from baseline or later NIHSS = 0), death or poor outcome (modified Rankin Scale [mRS] ≥4), and favorable outcome (mRS ≤2). We further evaluate statin withdrawal effects in cardioembolic stroke patients for these outcomes.

Among 443 IVT patients enrolled, 367 were included in the final study population. There were 88, 188, and 91 patients in the reference, continued, and withdrawal groups, respectively. Multivariable logistic regression showed that statin withdrawal compared with the reference was related to a lower possibility of long-term favorable outcome (OR = 0.45, 95% CI [0.22, 0.90], *P* = 0.024). Compared with the continued group, the adjusted OR of statin withdrawal was 0.40 (95% CI [0.22, 0.72], *P* = 0.002) and 2.52 (95% CI [1.34, 4.75], *P* = 0.004) for long-term favorable and poor/death outcomes, respectively. Also, results were similar for cardioembolic stroke patients (OR = 0.35, 95% CI [0.14, 0.89], *P* = 0.027 of favorable outcome and OR = 3.62, 95% CI [1.37, 9.62], *P* = 0.010 of poor/death outcome).

In a real-world setting, for stroke patients receiving intravenous thrombolysis, statin withdrawal within 3 weeks initiating in 72 hours may have a harmful effect on the long-term neurologic outcome, even in cardioembolic stroke patients.

## INTRODUCTION

Statins (3-hydroxyl-3-methylglutary coenzyme A reductase inhibitors) have clearly presented benefits by accumulating body of evidences for reducing the risk of primary and secondary strokes. In addition to their cholesterol-lowering effect, statins have also been proved to provide a pleiotropic non-cholesterol-dependent effects so as to reduce mortality and improve both short- and long-term outcomes in stroke patients.^1,2^ In clinical practice, statin therapy before and during stroke hospitalization was likely to lead to a good discharge outcome.^3^ Specially, statin withdrawal in acute stroke phase with prior statin treatment was found to be associated with an increased risk of dependency at 90 days, indicating that a rebound phenomenon may exist with statin cessation.^4^

Limited research about the combination of thrombolysis and statin therapy so far showed controversial results. A latest meta-analysis revealed that among thrombolytic patients, prestroke use of statin was associated with neither good outcome nor death at 90 days.^5^ However, another large-scale retrospective study, the THRombolysis and Statins (THRaST) study, showed that statin use in the acute phase of stroke after intravenous thrombolysis may improve neurologic outcome and decrease the mortality at 90 days.^6^ Noteworthy, the adherence of statin therapy after discharge was poorly assessed in these studies.

Considering the increasing application of intravenous rt-PA and statins, it is especially crucial to clarify whether the noncompliance of statin therapy after the acute phase will affect the neurological outcome of thrombolytic stroke patients. This current study is thus designed to investigate the impact of statin withdrawal within 3 weeks on the outcome of the thrombolytic patients who received statins in the acute phase.

## MATERIALS AND METHODS

### Study Subjects

This is a retrospective monocenter study based on data prospectively and continuously collected from June 2009 to May 2014. We then consecutively enrolled patients who had a diagnosis of acute ischemic stroke confirmed by diffusion-weighted imaging (DWI) or computer tomography perfusion imaging after informed consent; received intravenous recombinant tissue-type plasminogen activator (rt-PA), and intravenous rt-PA (alteplase 0.9 mg/kg up to a maximum of 90 mg) was used with 10% of the total dosage as a bolus and the rest over 1 hour. No patient received antithrombotic agents within 24 hours after rt-PA infusion.

Thrombolysis exclusion criteria were according to the Safe Implementation of Thrombolysis in Stroke-Monitoring Study (SITS-MOST) protocol,^7^ except for the 80-year age limit, onset to intravenous thrombolytic treatment (OTT) time for IV rt-PA from 3 to 4.5 hours from symptom onset, history of stroke and concomitant diabetes, and aggressive management (intravenous medication) to reduce blood pressure (systolic blood pressure ≤185 mm Hg or diastolic blood pressure ≤110 mm Hg) before intravenous rt-PA. The exclusion criteria for our statin withdrawal study were the same, besides prior use of statin, metastatic neoplasms, surgery such as craniectomy or mechanic embolectomy, dead or palliative care in 72 hours, follow-up uncompleted, and baseline modified Rankin Score (mRS) >2.

Patients were divided into 3 groups: the reference group (not received statin within 72 hours after stroke onset); the continued group (initiated statin within 72 hours after stroke onset and continued for at least 3 weeks); the withdrawal group (initiated statin within 72 hours after stroke onset but discontinued in 3 weeks). The selection of statin administration doses and types was at the discretion of the charged physician. Patients were treated in a primary stroke unit following the guidelines of the Study Group for Neurologic Diseases of the Chinese Medical Association. Standardized rehabilitation and physical therapy were applied when the patients were stable. Stroke subtype was classified as large-artery atherosclerosis, cardioembolism, small-vessel occlusion, other determined etiology, and undetermined etiology.^8^

All patients were followed up and assessed by phone or in clinic at 7 day, 1 , and 3 months after the onset including the statin regimen condition and the neurologic evaluation. All neuroimaging evaluation and neurofunction assessments were acquired prospectively by trained investigators who were blinded to the group assignments. In case of statin withdrawal, patients were asked to provide the dates of cessation as well as the reason.

### Clinical Characteristics of the Patients

Clinical characteristics of the patients included demographic findings; medical history such as history of atrial fibrillation, coronary heart disease, congestive heart failure, hypertension, diabetes mellitus, dyslipidemia, previous stroke or TIA, and smoker; onset-to-treatment time for intravenous rt-PA; medication prior to admission, which indicated the antiplatelet therapy; thrombolysis-related information including onset-to-treatment time, NIHSS on admission, dosage of rt-PA, and incidence of symptomatic intracerebral hemorrhage (sICH). The sICH was defined as local or remote parenchymal hematoma type 2 on the image scan obtained 24 to 36 hours after treatment, with NIHSS score increase of ≥4 points from baseline or the lowest value in the first 24 hours or leading to death^7^; baseline information including baseline international normalized ratio (INR), baseline blood glucose, systolic blood pressure, diastolic blood pressure, and creatinine, lipid profile within 48 hours after admission which consisted of total cholesterol, low-density lipoprotein-cholesterol (LDL-C), and high-density lipoprotein-cholesterol (HDL-C); etiology of stroke; time to withdraw statin for the withdrawal group; modified Rankin Score at 3 months; dosage and type of statin administrated during 3 weeks after the stroke onset.

### Outcomes

Our safety end point was poor/death outcome (mRS ≥4) at 3 months. The efficacy end points were NIHSS improvement ≥4 in 7 days and 1 month and favorable outcome (mRS ≤2) at 3 months.

### Statistical Analysis

Mean with standard deviation, medians with interquartile range, and percentages were used to describe the distribution of continuous and categorical variables, respectively. Baseline characteristics were compared among the reference, continued, and withdrawal groups by Fisher exact test for categorical variables, analysis of variance (ANOVA), and Kruskal–Wallis 1-way analysis for continuous variables. We evaluated the effect of statin withdrawal by calculating odds ratio (OR) with 2-sided 95% confidence interval (CI) for each end point. Variables with a probability value <0.1 in univariate analysis were determined unbalanced clinical characteristics. All the unbalanced clinical characteristics and established predictors (age, OTT time, baseline NIHSS score) were then enrolled in multivariate logistic regression analysis. To further determine the difference between statin withdrawal and continuous use, we then compared each end point by comparing the withdrawal group with the continued group. Finally, to identify this effect in cardioembolic stroke patients, we performed multivariate analysis to test each end point in this subtype of stroke. Statistical significance was set at *P* < 0.05. All statistical analyses were performed using the Statistical Package for Social Sciences v 17.0 for Windows (SPSS, Chicago, IL).

## RESULTS

**FIGURE 1:**
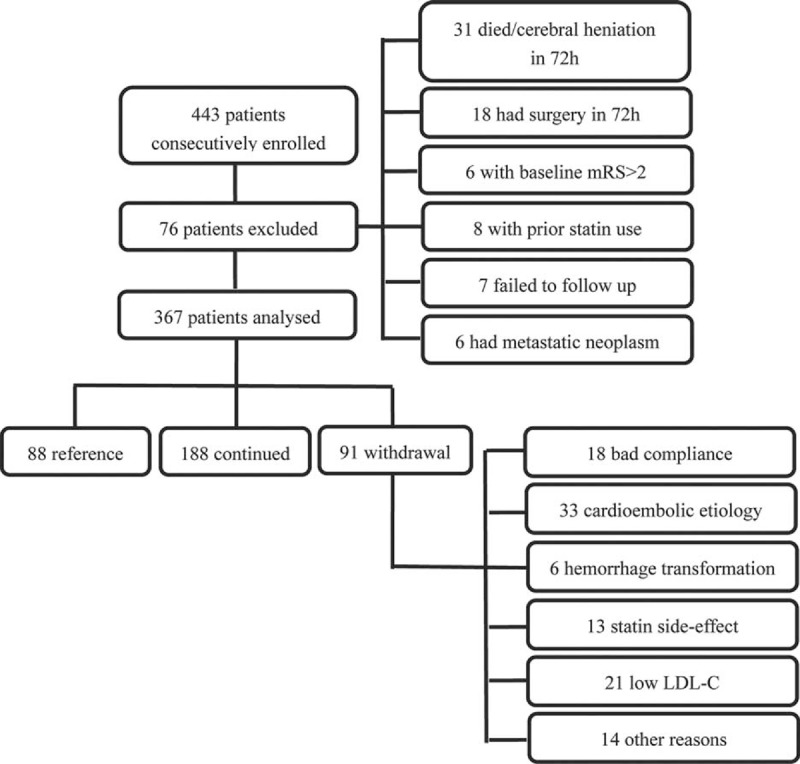
Study profile of patients’ inclusion and exclusion. LDL-C = low-density lipoprotein cholesterol.

**FIGURE 2:**
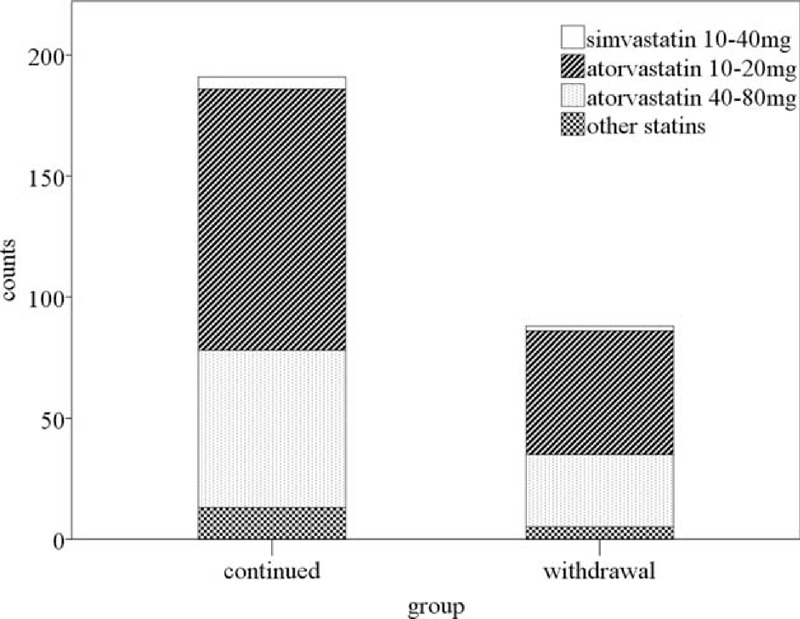
Statin regimen in continued and withdrawal groups.

A total of 443 patients were consecutively enrolled and received IVT during the period, among whom 76 patients were excluded (Figure 1). Finally, of the remaining 367 patients who were enrolled in the current study, 88 (24.0%) were in the reference group, 188 (51.2%) patients in the continued group, and 91 (24.8%) patients in the withdrawal group. In patients who received statins in 72 hours, the statin type and dose were atorvastatin 10 to 20 mg/day in 109 (58.0%) statin-continued patients and in 54 (59.3%) statin-withdrawal patients, atorvastatin 40 to 80 mg/day in 62 (33.0%) statin-continued patients and in 29 (31.9%) statin-withdrawal patients, simvastatin 20 to 40 mg/day in 4 (2.1%) statin-continued patients and in 3 (3.3%) statin-withdrawal patients, rosuvastatin 10 to 20 mg/day in 13 (6.9%) statin-continued patients and in 4 (4.4%) statin-withdrawal patients, pravastatin 20 mg in 1 (1.1%) statin-withdrawal patients. There was no significant distributional difference between the continued and withdrawal groups (Figure 2). The duration for statin treatment in the withdrawal group was 13.2 ± 5.7 days. Reasons for withdrawal of statin included bad compliance, cardioembolic as stroke subtype, discovery of hemorrhagic transformation after intravenous thrombolysis, presentation of side effect of statin, concerns of physicians about cerebral hemorrhage risk related to a rather low level of LDL-C, and unclarified reasons. Some patients discontinued statin therapy for 1 or more aforementioned reasons (Figure 1).

Table 1 showed the clinical characteristics of the reference, continued, and withdrawal groups. Baseline NIHSS was higher in the reference group than the other 2 groups. Atrial fibrillation and cardioembolic stroke were more frequent in the reference group, whereas higher frequency of large-artery atherosclerosis in the continued and withdrawal groups. The reference group also tended to have a lipid profile with lower total cholesterol, lower LDL-C, and higher HDL-C level, as well as a lower incidence to smoke.

**TABLE 1:**
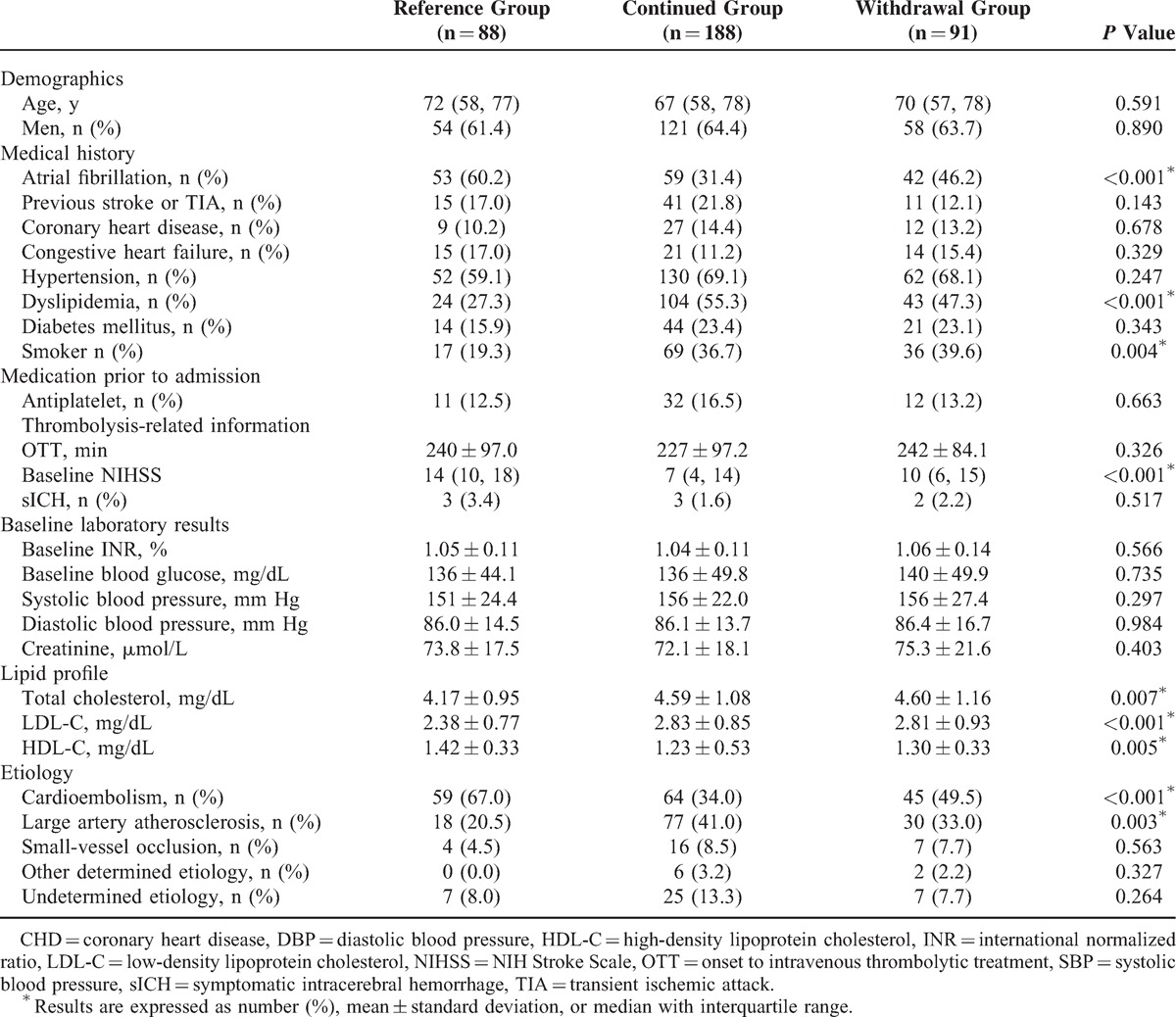
Baseline Clinical Characteristics in Patients Initiated Statin Within 72 Hours

The outcomes for short-, mid-, and long-term outcomes are shown in Table 2. The favorable outcome demonstrated a smaller probability to reach in the withdrawal group (OR = 0.45, 95% CI [0.22, 0.90], *P* = 0.024).

**TABLE 2:**
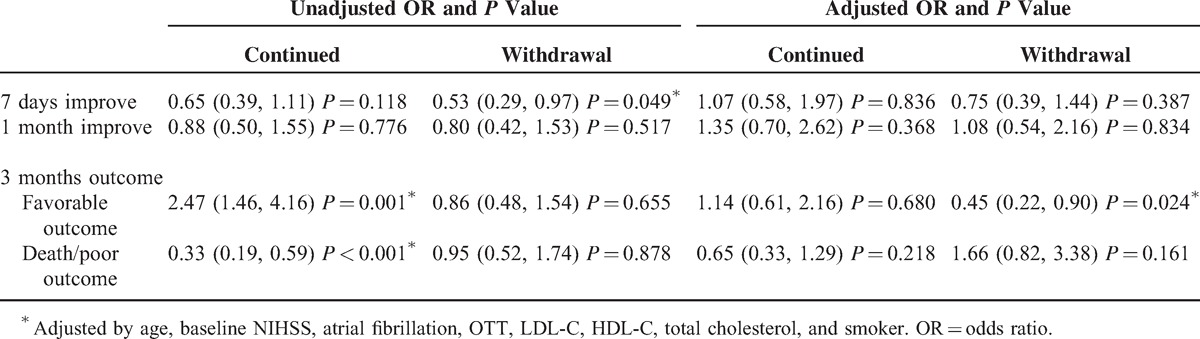
Unadjusted and Adjusted OR and *P* Value of Death or Poor Outcome and Favorable Outcome in Patients Who Initiated Statin in 72 Hours With or Without Statin Withdrawal Comparing to the Reference Group

Additionally, the same deleterious effect of statin withdrawal was disclosed by comparing with the continued group in Table 3. Statin withdrawal was associated with an OR of 0.40 (95% CI [0.22, 0.72], *P* = 0.002) for 3-month favorable outcome and an OR of 2.52 (95% CI [1.34, 4.75], *P* = 0.004) for 3-month poor outcome or death.

**TABLE 3:**
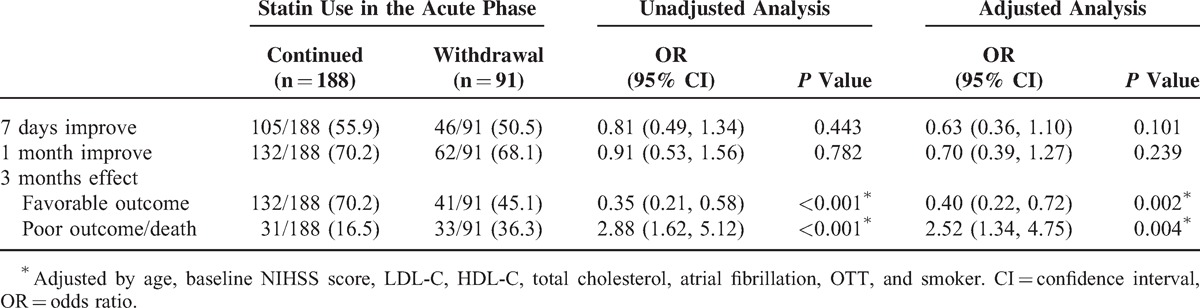
Univariate and Multivariate Analysis: Short-Term and Long-Term Effect of Statin Withdrawal Comparing to the Continued Group

Finally, we focused on the cardioembolic stroke subtype and found the difference of outcomes between the continued and withdrawal groups. As Table 4 exhibits, after adjusting the influences of age, baseline NIHSS, atrial fibrillation, LDL-C, HDL-C, total cholesterol, and smoking by multivariate logistic regression, favorable outcome defined as mRS ≤2 at 3 months was unlikely to emerge in the withdrawal group compared with the continued group (OR 0.35, 95% CI [0.14, 0.89], *P* = 0.027). Meanwhile, patients who withdrew statin therapy within 3 weeks were more likely to degenerate a poor outcome or death at 3 months (OR = 3.62, 95% CI [1.37, 9.62], *P* = 0.010).

**TABLE 4:**
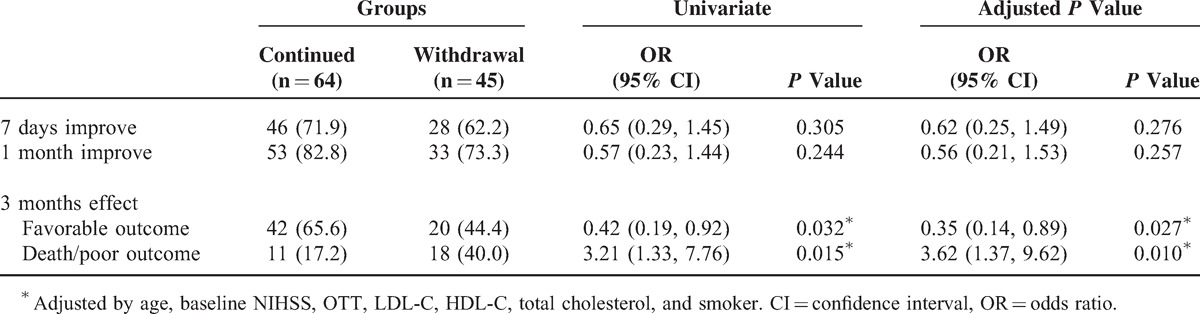
Univariate and Multivariate Analysis of Cardioembolic Stroke Patients: Short-, Mid-, and Long-Term Outcomes of Patients Who Initiated Statin in 72 Hours With or Without Statin Withdrawal

## DISCUSSION

The current study reveals that for patients receiving intravenous thrombolysis, statin withdrawal within 3 weeks when initiated in the acute phase (72 hours) was associated with a lower possibility to reach a long-term favorable outcome at 3 months in comparison with statin continuation. Furthermore, this negative correlation with favorable outcome was also shown when compared with those without initiation of statin in 72 hours.

The current study assesses the effect of statin withdrawal during after-discharge period in patients receiving intravenous thrombolysis, which has been rarely evaluated in previous studies.^4,6,9^ Our findings that discontinuation of statin therapy within 3 weeks was associated with a worsened long-term outcome, rather than short- or mid-term outcomes, are in line with studies in cardiovascular diseases.^10–12^ Given the prolonged deleterious effect of statin withdrawal, this result may be partly explained by the “rebound phenomenon.” As experimental models and clinical trials suggested, the benefits of previous statin therapy can be rapidly lost and result in a worsened outcome if statin was abruptly discontinued.^4,10,13,14^ Mechanisms underneath these findings were partially elucidated. To start with, statin discontinuation can suppress the endothelial nitric oxide (eNO) production, which was resulted from the increased activation of Rho guanosine triphosphatase protein, leading to the impairment of vascular homeostasis.^13^ Moreover, acute withdrawal of statin upregulated angiotensin II type1 (AT1) receptor in smooth muscle cells and exacerbated vascular dysfunction.^15^ Secondly, cessation of statin tended to untie the massive inflammatory response after stroke, which not only led to prolonged reelevated hs-CRP and interleukin-6,^16,17^ but also induced inhibition of angiogenic and reparative signaling on monocyte.^18^ Finally, in experimental models, discontinuation of statin was also assumed to reduce synaptogenesis as well as angiogenesis,^1,19^ which as a result haltered the cerebral endogenous recovery after stroke.^20^

A challenging finding in our study is that even in cardioembolic thrombolytic patients, withdrawal of statin therapy within 3 weeks was also correlated to a worse long-term outcome than the continued counterparts. Previous studies have suggested that the inflammatory response after ischemic stroke was irrespective of stroke subtypes, and cardioembolic subtype was even reported with higher level of interleukin-6, interleukin-1β, and tumor necrosis factor-α.^21^ Another possible explanation involved may be addressed as the comorbidity of atherosclerosis in cardioembolic patients.^22^ It is true since the comorbidity found in our study is nearly 50%, which was confirmed by vascular imaging such as vascular ultrasonography, computed tomography angiography, and magnetic resonance angiography. However, in this case we believe that the endogenous cerebral recovery independent with the atherosclerosis in these patients was more likely to be affected.

Importantly, statin has been demonstrated to have effects on the fibrinolysis and coagulation system by increasing the expression of t-PA and inhibiting the expression of plasminogen activator inhibitor type-1 (PAI-1),^23^ as well as by inhibiting platelet aggregation.^24^ Although concerning about increasing of transferred hematoma induced by combination of rt-PA and statins used to obstacle the clinical application, it is proved by numerous evidence that statin therapy is safe combined with rt-PA.^6,9,25^ In fact, it was reported that thrombolysis with exogenous rt-PA may lead to a delayed massive activation of coagulation factors and fibrin formation within the acute phase. For ischemic stroke patients, these rt-PA–induced blood abnormalities may lead to recurrent stroke after thrombolysis due to endogenous tPA inhibition.^26^ In this context, it is not surprising to find studies revealing that statin discontinuation not only ended the beneficial antithrombus impact but also promoted microthrombolus reform as well as inhibited collateral circulation.^27,28^

In a real-world setting, statin withdrawal now happens commonly rather after discharge than in-hospital for variant reasons.^27,29^ In the current study, we were able to assess the time and reasons to discontinue statin therapy as well as stroke subtype and detailed statin regimen. In this way, we further identified confounding factors responsible for statin withdrawal. Not surprisingly, patient compliance or cohesiveness seemed a critical factor to validate professional advices from doctors, but unfortunately, it was also usually neglected. Along with this, other researches also suggested home- and community-based education and rehabilitation were critical for a long-term outcome for patients who suffered cerebrovascular accident.^30^ Noteworthy though, in the present analysis of the withdrawal reasons, the bad compliance was not the dominant reason, while the unclear, subjective, and invalid discretion of physician and community physician seemed pivotal. Therefore, our findings may highlight the importance of health care providers’ perspectives.

We are aware of several limitations of this study. First of all, it was not a randomized controlled trial, thus inevitably there were several intergroup imbalances which may be related to unfavorable outcome after intravenous thrombolysis. High baseline NIHSS score and cardioembolic stroke subtype were more frequent in the withdrawal group than the continued group, which reflected that patients with higher severity of stroke tended to discontinue statin therapy. According to this, we tried to address these imbalances by adjusted multivariate analysis to diminish these imbalances. In addition, individuals in the withdrawal group may also result in a worse outcome due to lack of care for their own health which cannot be evaluated in our study.

In conclusion, our findings supported that statin initiated in the acute phase among thrombolytic stroke patients should not be interrupted within 3 weeks, even among patients with cardioembolic stroke. Large-scale trials with detailed observational data are necessary to reveal the long-term effect of statin for thrombolytic patients.

## References

[1] Chen J Zhang ZG Li Y Statins induce angiogenesis, neurogenesis, and synaptogenesis after stroke. *Ann Neurol* 2003; 53:743–751. 12783420 10.1002/ana.10555

[2] Fisher M Moonis M Neuroprotective effects of statins: evidence from preclinical and clinical studies. *Curr Treat Options Cardiovasc Med* 2012; 14:252–259. 22362392 10.1007/s11936-012-0174-9

[3] Flint AC Kamel H Navi BB Inpatient statin use predicts improved ischemic stroke discharge disposition. *Neurology* 2012; 78:1678–1683. 22614435 10.1212/WNL.0b013e3182575142

[4] Blanco M Nombela F Castellanos M Statin treatment withdrawal in ischemic stroke: a controlled randomized study. *Neurology* 2007; 69:904–910. 17724294 10.1212/01.wnl.0000269789.09277.47

[5] Chroinin DN Asplund K Asberg S Statin therapy and outcome after ischemic stroke systematic review and meta-analysis of observational studies and randomized trials. *Stroke* 2013; 44:448–456. 23287777 10.1161/STROKEAHA.112.668277

[6] Cappellari M Bovi P Moretto G The THRombolysis and STatins (THRaST) study. *Neurology* 2013; 80:655–661. 23345634 10.1212/WNL.0b013e318281cc83 PMC3590058

[7] Wahlgren N Ahmed N Davalos A Thrombolysis with alteplase for acute ischaemic stroke in the Safe Implementation of Thrombolysis in Stroke-Monitoring Study (SITS-MOST): an observational study. *Lancet* 2007; 369:275–282. 17258667 10.1016/S0140-6736(07)60149-4

[8] Adams HP Jr Bendixen BH Kappelle LJ Classification of subtype of acute ischemic stroke. Definitions for use in a multicenter clinical trial. TOAST. Trial of Org 10172 in Acute Stroke Treatment. *Stroke* 1993; 24:35–41. 7678184 10.1161/01.str.24.1.35

[9] Engelter ST Soinne L Ringleb P IV thrombolysis and statins. *Neurology* 2011; 77:888–895. 21849650 10.1212/WNL.0b013e31822c9135

[10] Daskalopoulou SS Delaney JA Filion KB Discontinuation of statin therapy following an acute myocardial infarction: a population-based study. *Eur Heart J* 2008; 29:2083–2091. 18664465 10.1093/eurheartj/ehn346

[11] Gomez Sandoval YH Braganza MV Daskalopoulou SS Statin discontinuation in high-risk patients: a systematic review of the evidence. *Curr Pharm Des* 2011; 17:3669–3689. 22074437 10.2174/138161211798220891

[12] Kim MC Cho JY Jeong HC Impact of postdischarge statin withdrawal on long-term outcomes in patients with acute myocardial infarction. *Am J Cardiol* 2015; 115:1–7. 25456863 10.1016/j.amjcard.2014.09.039

[13] Laufs U Endres M Custodis F Suppression of endothelial nitric oxide production after withdrawal of statin treatment is mediated by negative feedback regulation of rho GTPase gene transcription. *Circulation* 2000; 102:3104–3110. 11120702 10.1161/01.cir.102.25.3104

[14] Heeschen C Hamm CW Laufs U Withdrawal of statins increases event rates in patients with acute coronary syndromes. *Circulation* 2002; 105:1446–1452. 11914253 10.1161/01.cir.0000012530.68333.c8

[15] Castejon AM Zollner E Tristano AG Upregulation of angiotensin II-AT1 receptors during statin withdrawal in vascular smooth muscle cells. *J Cardiovasc Pharmacol* 2007; 50:708–711. 18091590 10.1097/FJC.0b013e318157c0b2

[16] Li JJ Li YS Chu JM Changes of plasma inflammatory markers after withdrawal of statin therapy in patients with hyperlipidemia. *Clinica Chim Acta* 2006; 366:269–273. 10.1016/j.cca.2005.10.021 16343471

[17] Li JJ Inflammatory rebound phenomenon after abrupt withdrawal of statin is a mature point of view but not hypotheses. *Int J Cardiol* 2011; 151:120. 21703704 10.1016/j.ijcard.2011.06.028

[18] Jaipersad AS Shantsila E Blann A The effect of statin therapy withdrawal on monocyte subsets. *Eur J Clin Invest* 2013; 43:1307–1313. 24134608 10.1111/eci.12183

[19] Teixeira MZ Statins withdrawal, vascular complications, rebound effect and similitude. *Homeopathy* 2010; 99:255–262. 20970095 10.1016/j.homp.2010.01.001

[20] Hermann DM Chopp M Promoting brain remodelling and plasticity for stroke recovery: therapeutic promise and potential pitfalls of clinical translation. *Lancet Neurol* 2012; 11:369–380. 22441198 10.1016/S1474-4422(12)70039-X PMC3964179

[21] Tuttolomondo A Di Raimondo D Pecoraro R Inflammation in ischemic stroke subtypes. *Curr Pharm Des* 2012; 18:4289–4310. 22390641 10.2174/138161212802481200

[22] Ohira T Shahar E Iso H Carotid artery wall thickness and risk of stroke subtypes: the atherosclerosis risk in communities study. *Stroke* 2011; 42:397–403. 21164133 10.1161/STROKEAHA.110.592261 PMC3026889

[23] Bourcier T Libby P HMG CoA reductase inhibitors reduce plasminogen activator inhibitor-1 expression by human vascular smooth muscle and endothelial cells. *Arterioscler Thromb Vasc Biol* 2000; 20:556–562. 10669656 10.1161/01.atv.20.2.556

[24] Tsai NW Lin TK Chang WN Statin pre-treatment is associated with lower platelet activity and favorable outcome in patients with acute non-cardio-embolic ischemic stroke. *Crit Care* 2011; 15. 10.1186/cc10303 PMC3387600 21740551

[25] Campos M Garcia-Bonilla L Hernandez-Guillamon M Combining statins with tissue plasminogen activator treatment after experimental and human stroke: a safety study on hemorrhagic transformation. *CNS Neurosci Ther* 2013; 19:863–870. 24118905 10.1111/cns.12181 PMC6493385

[26] Fassbender K Dempfle CE Mielke O Changes in coagulation and fibrinolysis markers in acute ischemic stroke treated with recombinant tissue plasminogen activator. *Stroke* 1999; 30:2101–2104. 10512913

[27] Cubeddu LX Seamon MJ Statin withdrawal: clinical implications and molecular mechanisms. *Pharmacotherapy* 2006; 26:1288–1296. 16945051 10.1592/phco.26.9.1288

[28] Lai WT Lee KT Chu CS Influence of withdrawal of statin treatment on proinflammatory response and fibrinolytic activity in humans: an effect independent on cholesterol elevation. *Int J Cardiol* 2005; 98:459–464. 15708180 10.1016/j.ijcard.2003.11.023

[29] Daskalopoulou SS When statin therapy stops: implications for the patient. *Curr Opin Cardiol* 2009; 24:454–460. 19574923 10.1097/HCO.0b013e32832ebf92

[30] Altman IM Swick S Malec JF Effectiveness of home- and community-based rehabilitation in a large cohort of patients disabled by cerebrovascular accident: evidence of a dose-response relationship. *Arch Phys Med Rehabil* 2013; 94:1837–1841. 23462581 10.1016/j.apmr.2013.02.014

